# How does Internet use impact the mental health of social assistance recipients in China? Examining the chain mediating role of social support and family resilience

**DOI:** 10.3389/fpsyg.2024.1460064

**Published:** 2025-01-06

**Authors:** Bin Wu, Beihai Tian

**Affiliations:** Rural Construction and Management Research Center, Huazhong Agricultural University, Wuhan, China

**Keywords:** Internet use, mental health, social support, family resilience, social assistance recipients, Mindsponge Theory, COR Theory

## Abstract

**Introduction:**

Previous studies have explored the relationship between Internet use and mental health, but there has been a lack of focus on social assistance recipients. Additionally, there has been insufficient discussion on the impact mechanisms of social support and family resilience on this relationship. This study aims to fill these gaps. Using data from social assistance recipients in China, this study analyzes the relationship between Internet use and the mental health of social assistance recipients and its mechanisms based on the Mindsponge Theory and Conservation of Resources Theory.

**Methods:**

This study used multiple linear regression models and PROCESS models to complete data analysis on 765 Chinese social assistance recipients samples to test the hypothesis. All analyzes were performed using SPSS26.0 and MPlus 8.0.

**Results:**

The empirical analysis shows that Internet use positively correlates with the mental health of social assistance recipients, and social support and family resilience mediate between Internet use and mental health. In addition, social support and family resilience also play a chain mediating role in the relationship between Internet use and mental health.

**Discussion:**

The analysis results of this study support our hypothesis. Combined with the findings of this study, we discussed the theoretical significance, practical significance, limitations, and future research directions of this study.

## Introduction

By the end of 2020, China had successfully lifted all its poor people out of poverty under the established standards (World Bank, 2022), taking a historic step toward global poverty reduction and development. Behind this achievement, China’s social assistance policy has played a vital role. However, while the basic living needs of China’s social assistance recipients are currently guaranteed, China’s social assistance policy is still mainly based on material assistance (Zhang, 2014), including cash and daily necessities. Service-oriented social assistance that meets the diverse needs of social assistance recipients, such as psychological counseling and life care, is not sufficiently developed. For social assistance families with special difficulties, their spiritual needs for assistance, such as life guidance, psychological comfort, and social integration, have not yet been fully met. In general, the mental health of social assistance recipients in China has not received enough attention.

In recent years, scholars have conducted research on mental health issues, with the research subjects mainly focusing on the elderly (Grolli et al., 2021; Cheng et al., 2023; Kasenzu, 2024), children, adolescents (Meherali et al., 2021; Caamaño-Navarrete et al., 2024; Sheng and Motevalli, 2024), young adults (Coulaud et al., 2024), and the general population (Cullen et al., 2020; Talevi et al., 2020; Kupcova et al., 2023). Research on social assistance recipients (Malmberg-Heimonen, 2011; Scholten et al., 2023), especially those in China (Qi and Wu, 2018), is relatively insufficient. According to the results of the China Family Tracking Survey, about 7.23 percent of social assistance recipients in China suffer from serious mental illness (Liu, 2020). Compared with the general population, the mental health of the recipients of social assistance is significantly worse. Even if they receive social assistance and play its “buffer” role under stressful life events, their mental health is still in a poor state.

In terms of influencing factors of mental health, many studies have explored the relationship between Internet use and mental health and its mechanism. In the context of increasing Internet use, numerous empirical studies have shown that Internet use significantly improves people’s mental health (Yuan, 2021; Zhang and Zhang, 2021; Ding et al., 2022). For example, Internet use can reduce the degree of psychological depression, and social alienation (Xu and Zhang, 2023) and enhance psychological welfare (Forsman and Nordmyr, 2017). Based on exploring the relationship between Internet use and mental health, many scholars have explored its impact mechanism and found that Internet use can enhance people’s mental health by increasing the frequency of social interactions (Chen et al., 2023), strengthening social capital (Zhu et al., 2021), and improving life satisfaction (Zhang et al., 2022). In addition, some studies have produced different results, indicating that the increase in Internet use also brings the risk of Internet addiction, which is detrimental to people’s mental health (Choi et al., 2017; Wu et al., 2023).

Besides the above influencing factors, some scholars have also explored the important influence of family resilience and social support on individual mental health. Studies have found that higher family resilience is significantly associated with lower symptoms of anxiety, stress, and depression (Gomez, 2021; Zhuo et al., 2022). Adequate social support and good coping strategies have a positive impact on family resilience during the pandemic (Gayatri and Irawaty, 2022), and social support can have direct and indirect positive effects on the mental health of family members by enhancing family resilience (Cao et al., 2020).

Although there has been much discussion of the relationship between Internet use and mental health in existing research, to the best of our knowledge, little attention has been paid to the possible chain-mediated role of social support and family resilience in this relationship, as well as the mental health issues of Chinese social assistance recipients. According to the latest data from the China Internet Network Information Center (2024), China has experienced rapid development of the Internet in recent years. By December 2023, the number of Internet users in China is 1.092 billion, and the Internet penetration rate has reached 77.5%, of which 99.9% use mobile phones to access the Internet, and the number of users using instant messaging, online video, and online shopping has reached 1.060 billion, 1.067 billion, and 915 million, respectively. In this context, it is necessary to clarify the relationship and mechanisms between Internet use and the mental health of social assistance recipients.

This study is based on the Mindsponge Theory and the Conservation of Resources (COR) Theory to explain the relationship between Internet use and mental health. On the one hand, people’s mental health is often closely related to their social interactions with other people and the surrounding environment, and these social interactions can be regarded as an information process. According to the Mindsponge Theory, the information process may play an important role in shaping people’s core values and influencing people’s mental health. Realizing this process is inseparable from information accessibility and information screening mechanism based on cost–benefit judgment (Nguyen et al., 2021). The greater the accessibility of information, the more opportunities people have to integrate information through the information screening mechanism, and the greater the possibility of updating people’s core values (Vuong et al., 2022b), thus affecting their mental health. On the other hand, the COR Theory holds that when people face the risk of resource loss or have incurred actual losses, they will have a stress response, making individuals more susceptible to mental health problems such as depression and anxiety (Hobfoll, 1989). People with fewer resources are more likely to suffer from chronic and severe stress. In the case of resource loss, the replenishment and increase of resources is very important (Hobfoll et al., 2018), which can play a significant role in alleviating stress and anxiety, thus contributing to mental health.

Social assistance recipients are different from the general group. Most of them experience short-term or long-term difficulties, such as encountering accidents, family members suffering from serious illnesses, etc. These experiences often lead to huge losses of various resources, while still facing the risk of continuous loss of resources to a certain extent, which also brings greater perceived costs to overcome difficulties. As an important means for people to obtain information and resources, Internet use can, on the one hand, improve the accessibility of social assistance recipients to various potential social support information, reduce their perceived costs of overcoming crises, and thus help them internalize a more positive attitude toward coping with adversity, enhance their family resilience, and improve their mental health. On the other hand, Internet use can also help social assistance recipients obtain more resources from relatives, friends, significant others, communities, and society, thereby enhancing their perceived social support, improving their family resilience, and ultimately relieving stress and improving mental health.

**Figure 1:**
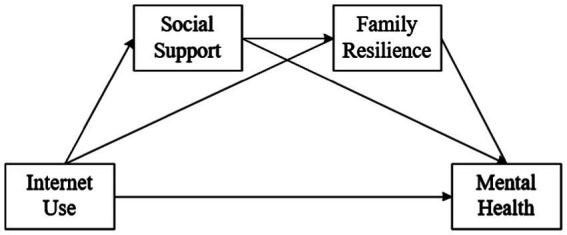
The conceptual model of this study.

In summary, this study has two contributions to the literature on Internet use and mental health. First, existing studies pay little attention to the mental health of Chinese social assistance recipients, and the relationship between Internet use and the mental health of Chinese social assistance recipients needs to be explored. To fill this gap, this study uses social assistance recipients as the research subjects to test whether there is a positive correlation between Internet use and their mental health. Second, by testing the chain mediation effect of social support and family resilience, the mechanism of the relationship between Internet use and the mental health of social assistance recipients is revealed. The conceptual model is shown in Figure 1.

## Literature review

### Direct effect of Internet use on mental health

Mental health is an integral part of overall health and is just as important as physical health. The World Health Organization defines mental health as a healthy mental state that helps people cope with stress and difficulties, actively learn and work, give full play to their abilities, and contribute to the community (World Health Organization, 2022). In research, scales related to anxiety, depression, and other psychological symptoms are often used to evaluate the positive and negative dimensions related to people’s mental health levels (Xu et al., 2011).

Internet use is an important skill for people to work and live, including social interaction, entertainment, learning, shopping, work, etc. Internet use can meet people’s various needs, thereby improving people’s psychological well-being (Wu et al., 2023).

To clarify the relationship between Internet use and the mental health of social assistance recipients, the Mindsponge Theory and the COR Theory were used in this study. The Mindsponge Theory was proposed by Vuong and Napier (2015). This theory assumes that everyone has their core values, and these values have an important influence on their psychological processes. It reveals people’s information processing mechanism for receiving and screening information, that is, people selectively absorb information based on their situations to form new values and abandon inappropriate values. Information from the environment needs two conditions to be accepted and integrated, including the accessibility of information (Nguyen et al., 2022) and a good evaluation of the information (Vuong et al., 2022b). Firstly, the accessibility of information requires that there is accessible information in the environment around people. Secondly, when the information in the environment is received, it needs to be screened and evaluated by multiple filtering systems. This screening process is mainly based on cost-effectiveness judgments and is influenced by people’s core values and situations (Nguyen et al., 2021). When people believe that the benefits of absorbing this information are greater than the cost, they will integrate this information into their existing values and affect their psychological processes, otherwise, they will exclude it.

The COR Theory was proposed by Hobfoll (1989), which attempts to explain the stress people face with a new resource-oriented model. It defines resources as objects, personal traits, conditions, or energies that individuals or groups value, or the means to achieve and obtain these objects, personal traits, conditions, and energies. One of the main assumptions of the COR Theory is that people will strive to preserve, protect, and build various resources, and the potential or actual loss of resources will pose a threat to people (Hobfoll et al., 2018). Furthermore, when people face the risk of resource loss or actual resource loss, it brings stress and anxiety to people, which is more likely to affect mental health. Therefore, when faced with the risk of resource loss or actual resource loss, people will restore and increase resources in different ways to help them cope with difficulties, relieve stress, and thus improve mental health.

Along these lines, social assistance recipients experience actual resource losses in dealing with family risk events. When their Internet use frequency is higher, they can obtain more information and resources through the Internet, which may help them relieve stress. Therefore, it may be associated with a higher level of mental health. On the one hand, Internet use increases their accessibility to various information that helps them cope with difficulties. At the same time, their disadvantaged status increases their perceived cost of getting out of trouble and their need for help, making them tend to absorb this information after judging the cost–benefit of this information, regard them as effective sources of help, and exclude information related to negative psychological states such as anxiety about their situation, so their mental health level may be higher. On the other hand, increased Internet use can help social assistance recipients alleviate the resource loss caused by risk events, thereby reducing stress reactions and helping to maintain a higher level of mental health. Therefore, we can make the following hypothesis:

*H1*. Internet use is positively correlated with mental health.

### Mediating effect of social support

Social support is a relatively broad concept. Wills (1991) defined social support as a person’s perception or experience of being cared for and respected by others (partners, relatives, friends, colleagues, and other social relations), and it is also part of a social network centered on mutual assistance and obligations. Specifically, it includes three types: informational support (helping the other party understand stressful events and feasible strategies), instrumental support (providing practical assistance and resources), and emotional support (providing spiritual comforts such as care and love). Barrera (1986) divides social support into actual social support and perceived social support. Actual social support refers to the clear help or resources obtained from the other party. Perceived social support refers to people’s belief that such resources or help can be obtained from social relationships when they are in trouble, that is, the individual’s subjective judgment on the degree of support they receive from the outside world.

In terms of research on the relationship between social support and mental health, there are two main views, namely the “main effect” and the “buffer effect.” On the one hand, the “main effect” suggests that social support has an important effect on people’s mental health, whether they experience a stressful event or not (Warheit, 1979; House et al., 1988). On the other hand, the “buffer effect” holds that social support has a significant positive effect on mental health only when people experience stressful events (Frydman, 1981; Bell et al., 1982). Both viewpoints believe that there is a significant relationship between social support and mental health. Some research further pointed out that emotional support is the most important dimension of social support, and this perceived social support has an important impact on mental health (Berkman and Kawachi, 2000). In fact, perceived social support is more important than actual received social support, and the latter can promote people’s psychological adjustment through the former (Wethington and Kessler, 1986). Based on these views, we mainly focus on perceived social support in our study.

From the perspective of the Mindsponge Theory (Nguyen et al., 2022), information accessibility is an important prerequisite for people to continuously update their core value system through the information process. When people are in trouble, Internet use can significantly improve people’s information accessibility by strengthening their social connections, which creates favorable conditions for them to obtain and absorb more social support information. At the same time, considering the predicament, in the cost–benefit judgment of this social support information, the perceived benefits of absorbing information are often far greater than its costs, so they are more likely to choose to integrate them into their core values, thereby enhancing perceived social support. For example, when social assistance recipients encounter risky accidents, they may increase the frequency of Internet use to strengthen their connections with relatives, colleagues, friends, and communities to obtain more information, resources, and emotional support. Through this process, they may feel the care and support from others, thereby gaining spiritual comfort, in other words, enhancing their perceived social support (Zhu et al., 2021). At the same time, existing research also shows that the stronger people’s perceived social support, the higher their mental health level (Guruge et al., 2015; Ozen et al., 2021; Zhu, 2024). Therefore, social support may play a mediating role between Internet use and mental health. Consequently, we propose Hypothesis 2:

*H2*. Social support mediates the relationship between Internet use and mental health.

### Mediating effect of family resilience

Family resilience is one of the important concepts in family system research in recent years. It attempts to explain why some families can maintain a better living condition and face difficulties with a positive attitude and actions when facing difficulties and pressures, while other families fail to cope with crises well and cause more conflicts. McCubbin and McCubbin (1988) identified family resilience as the characteristics, dimensions, and attributes of the family, that help the family maintain resilience in the face of crises, successfully overcome difficulties, and restore normal functions. Walsh (2016a,b) further proposed a theoretical model of the family resilience system which includes three key processes: the family belief system, the family organization model, and the family communication process. Specifically, the family belief system helps the family face difficulties, respond with a positive attitude, and strengthen the power of faith. The family organization model refers to the family maintaining flexibility and connectivity and mobilizing various social resources to enhance the ability to resist risks. The family communication process forms a joint force among family members through the communication of mutual trust, fully respecting individual differences and freedom of emotional expression. In general, family resilience reflects the family’s perception of risk and its ability to alleviate difficulties through communication and resource mobilization. Developing family resilience requires not only good communication, mutual respect, and the belief in actively coping with difficulties among family members, but also sufficient available resources.

From the perspective of COR Theory (Hobfoll, 1989), family resilience can be regarded as a favorable condition for individuals to cope with stressful situations, contains important resilience energy, can help family members alleviate the tension and anxiety caused by risks, and maintain a positive attitude to face difficulties together. Therefore, the improvement of family resilience may be beneficial to the mental health of family members. Some studies have also confirmed that higher family resilience is associated with lower anxiety, stress, and depression symptoms (Gayatri and Irawaty, 2022). In addition, family resilience, as an ability to solve problems by mobilizing resources, requires necessary resources. Internet use, as an important way to enhance social connections and obtain resources and information, can help recipients of social assistance in distress obtain more resources, laying the foundation for development, thereby helping to enhance their family resilience. In conclusion, we suggest that family resilience may act as a mediator between Internet use and the mental health of social assistance recipients. This leads us to hypothesis 3:

*H3*. Family resilience mediates the relationship between Internet use and mental health.

### Chain mediation effect of social support and family resilience

In the above discussion, we mentioned that the operation of family resilience requires not only the cooperation and communication of family members but also the contact of family members with the outside to obtain resources and support (Walsh, 2016a). In other words, perceived social support through important social relations such as relatives, friends, and co-workers may enhance families’ confidence and ability to cope with adversity. A positive relationship may exist between perceived social support and family resilience. Meanwhile, based on the Mindsponge Theory (Vuong et al., 2022a), after absorbing various social support information through benefit–cost evaluation, people’s perceived level of social support will be improved, and these new values will be integrated into the core values of social assistance recipients. Therefore, people will be able to face difficulties with a more positive attitude, form a correct risk perception, unite the strength of family members, and mobilize various social support resources to help the family through the crisis. In other words, perceived social support actually improves the family’s resilience level, promotes the family’s recovery and development, and thus helps social assistance recipients maintain good mental health. Relevant empirical studies have also shown that social support can enhance family resilience (Cassidy and Barnes, 2012), and social support provided by family members can enhance the ability of families to regain control (Wong et al., 2019).

Furthermore, based on the above hypothesis, we assume that Internet use may be positively correlated with perceived social support, while family resilience may be positively correlated with the level of mental health of social assistance recipients. Accordingly, it can be speculated that, first, there may be a positive correlation between the Internet use of social assistance recipients and their perceived social support. Second, as the perceived social support increases, the family resilience may tend to increase as well. Finally, family resilience may be positively correlated with the mental health of social assistance recipients. In summary, we propose the following hypothesis:

*H4*. Social support and family resilience act as chain mediators between Internet use and mental health.

## Materials and methods

### Sampling

The data for this study comes from the research project “Research on the Issue of Confirming Social Assistance Recipients through the Informed Commitment System” commissioned by the Department of Civil Affairs of Hubei Province, China, to Huazhong Agricultural University. The questionnaire survey was conducted in Hubei Province from September to October 2021. The questionnaire mainly includes 11 modules, namely “basic family information,” “family assets, life, income and consumption,” “disaster/hardship experience and social support,” “resilience,” “social security,” “dishonest behavior and punishment of social assistance recipients,” “public services and social participation,” “social networks and information acquisition,” “functional activities and opportunities,” “subjective attitudes and evaluations,” and “employment and work.” The survey subjects were mainly families of social assistance recipients and families of non-social assistance recipients in the same community. The survey adopted a stratified sampling method. The first step was to select Yichang City and Huangshi City in Hubei Province based on the level of economic development and the implementation of relevant policies. The second step was to select 3 counties (districts) in each city. The third step was to select 4 to 5 towns or streets in each county (district). The fourth step was to select 2 villages (communities) in each town and street for household surveys. Finally, 773 samples of social assistance recipients and 407 samples of non-assistance recipients were obtained. After data cleaning and variable screening, 765 samples of social assistance recipients were included in the data analysis.

### Measurement

Considering that the mental health, social support, and family resilience scales used in this study are all English scales, we first translated these scales into Chinese. Specifically, the research team members first translated all the items of the original scales, then compared and analyzed the translated manuscripts, and repeatedly revised each sentence to form the first draft of the Chinese version of the scale. Secondly, two professors were invited to make revisions to the first draft, and the first draft was revised accordingly to make it more accurate and in line with the Chinese context. Finally, 20 respondents were invited to conduct a pre-survey. All the respondents were able to complete the scales and understand the meaning of each item. Based on the feedback from the pre-survey, the research team debugged and improved the scales again to form the final Chinese version of the mental health, social support and family resilience scales. In addition, to ensure the reliability and validity of the measurement results of the three scales, this study also conducted reliability and validity tests on the three scales. The results are shown in Table 1.

**Table 1:** Reliability and validity test of the scale and confirmatory factor analysis properties.

Construct	Factor loadings	CR	AVE
**Family Resilience**			
**Family belief system**			
My family can face difficulties together as a whole	0.831^***^	0.973	0.769
It is a common phenomenon that every family will encounter difficulties	0.758^***^		
My family sees difficulties as a challenge to be met and overcome by working together	0.885^***^		
My family tried to sort through the dilemma and focus on our choices and decisions	0.925^***^		
My family is full of hope and confidence to overcome the difficulties	0.900^***^		
My family encourages each other to build on our strengths	0.928^***^		
My family seized the opportunity, took action, and worked for it	0.929^***^		
My family focuses on what we can do and accepts the things we cannot change	0.902^***^		
My family shared common values and life goals	0.901^***^		
The process of coping with difficulties stimulates creativity and strengthens family bonds	0.830^***^		
Difficult experiences have increased my family’s compassion and desire to help	0.842^***^		
My family believes in learning from difficult situations and becoming stronger	0.831^***^		
**Family organization and communication process**			
My family is able to flexibly adapt to new challenges	0.812^***^	0.982	0.756
Family members can share stress and anxiety with each other	0.895^***^		
The leadership of our family makes us feel safe and secure	0.840^***^		
Families can be counted on to help each other in times of trouble	0.887^***^		
My family respects our individual needs and differences	0.892^***^		
In the immediate family and extended family, we have role models and reliance	0.799^***^		
My family can count on the support of friends and the village (community)	0.732^***^		
We can make use of the resources in the village (community) to help our family through difficult times	0.727^***^		
We try to make sense of our difficulties and possible solutions	0.846^***^		
Our family members are able to match their words with their actions	0.886^***^		
In our family, we can express our opinions and be honest with each other	0.917^***^		
Negative emotions (e.g., sadness, anger, fear) can be shared at home.	0.891^***^		
Families understand each other and avoid blaming each other	0.914^***^		
When we are in trouble, we can share positive emotions and relieve the anxiety caused by difficulties	0.935^***^		
My family discusses and makes decisions together and handles disagreements fairly	0.937^***^		
My family focuses on our goal and takes steps to achieve it	0.936^***^		
My family celebrates success and learns from mistakes together	0.900^***^		
Family members plan and prepare for the future and try to prevent the crisis from happening again	0.869^***^		
**Social support**			
**Support from family and significant others**			
There are people (bosses, relatives, colleagues) who are there for me when I have a problem	0.766^***^	0.947	0.691
I can share happiness and sorrow with some people (leaders, relatives, colleagues)	0.823^***^		
My family can help me in a concrete way	0.828^***^		
I can get emotional help and support from my family when needed	0.855^***^		
Some people (leaders, relatives, colleagues) are a real source of comfort when I am in trouble	0.855^***^		
I can talk to my family about my problems	0.835^***^		
There are people in my life (leaders, relatives, colleagues) who care about my feelings	0.829^***^		
My family is willing to help me make all kinds of decisions	0.853^***^		
**Support from friends**			
My friends can really help me	0.919^***^	0.960	0.856
I can count on my friends in times of trouble	0.929^***^		
My friends can share happiness and sorrow with me	0.933^***^		
I can discuss my problems with my friends	0.920^***^		
**Mental health**			
**Positive self-identity**			
I think I’m still useful	0.786^***^	0.756	0.513
I can handle my daily affairs well	0.773^***^		
I feel like I have something to live for	0.569^***^		
**Fear and anxiety**			
I tend to get nervous and anxious	0.888^***^	0.927	0.810
I tend to get upset or frightened	0.960^***^		
I tend to get frustrated or depressed	0.848^***^		

Standardized coefficients reported; ^***^p < 0.01; AVE, Average variance extracted; CR, Composite reliability.

#### Mental health

The mental health variable of this study was measured by the mental health scale designed by the research team in the questionnaire. The scale selected some items from the Self-Rating Anxiety Scale (SAS) and the Self-Rating Depression Scale (SDS), including 6 items such as “I tend to get frustrated or depressed “and “I think I am still very useful “(Zung, 1965, 1971). Each question contains 5 options: “strongly disagree “, “disagree “, “undecided “, “agree “and “strongly agree “, with values of 1–5, respectively. After completing the principal component analysis of the scale, two common factors were extracted, named the “positive self-identity” factor (including three items “I think I am still very useful,” “I can handle my daily affairs well” and “I feel like I have something to live for”) and the “fear and anxiety “factor (including three items “I tend to get nervous and anxious,” “I tend to get upset or frightened” and “I tend to get frustrated or depressed”). Then the two factors were used to calculate the comprehensive score, and the score was further converted into 1–100 to measure the mental health level of the social assistance recipients (*M* = 61.130, SD = 17.515). Cronbach’s alpha for this scale was 0.785.

#### Internet use

In our study, we mainly discuss the frequency of Internet use. Respondents were asked how often they participated in a series of Internet use activities in the past year, including chatting, entertainment, browsing news, studying, working, and online shopping. Each item contains six options and is assigned a value from 0 to 5 (0 = “never”; 1 = “several times a year”; 2 = “at least once a month”; 3 = “at least once a week”; 4 = “several times a week”; 5 = “almost every day”). These scores were added and averaged to measure the frequency of Internet use (*M* = 1.295, SD = 1.429).

#### Social support

The Perceived Social Support Scale (PSSS) was used to measure the level of social support for the social assistance recipients in our study (Zimet et al., 1988). Respondents were asked to rate their level of agreement in 12 statements based on their situations. Each statement had 5 options, with values of 1–5 (1 = “strongly disagree” to 5 = “strongly agree”). After principal component analysis, two common factors were extracted, namely “support from family and significant others” and “support from friends.” The two factors were also used to calculate the comprehensive score and convert it into a score of 1–100, which was used to measure the level of social support of social assistance recipients. Cronbach’s alpha for this scale was 0.954.

#### Family resilience

Respondents were asked to complete a 30-item scale to measure their family resilience score. These items are derived from the Walsh Family Resilience Questionnaire (Walsh, 2016b), and are screened and adjusted according to the Chinese context. Each question contains 5 options and is assigned a score of 1–5 (1 = “strongly disagree” to 5 = “strongly agree”). We first used factor analysis on this scale and extracted two factors, named “Family belief system” and “Family organization and communication process.” Second, we get a score from 1 to 100 in the same way as the variables above. Finally, we used this score to evaluate the family resilience of social assistance recipients (*M* = 66.245, SD = 21.202). The reliability for the scale as indicated by Cronbach’s *α* was remarkably high at 0.988.

#### Control variables

Control variables included 3 sets of variables: demographic variables, average family income, and social contact. The demographic variables included gender (0 = “female”; 1 = “male”), age (year), physical health status (1 = “unhealthy” to 5 = “very healthy”), marriage status (four dummy variables are generated according to the four options of “single,” “married,” “widowed,” and “divorced.” “Single” is taken as the reference group), education level (0 = “uneducated,” 1 = “graduated from literacy class/did not graduate from primary school”; 2 = “graduated from primary school”; 3 = “graduated from junior high school,” 4 = “graduated from high school/technical school,” 5 = “graduated from junior college,” 6 = “bachelor degree or above”), politics status (0 = “Non-CPC member”;1 = “CPC member”), and current residence (0 = “rural area”; 1 = “urban area”). Average family income was measured by the natural logarithm of the average income of its members. Social contact was measured by the respondents’ interaction frequency scores with friends, neighbors, and community cadres (1 = “never,” 2 = “several times a year,” 3 = “at least once a month,” 4 = “at least once a week,” 5 = “several times a week”). The average of the three scores was then used to measure the social contact level of the social assistance recipients.

### Data analysis

In our study, the four core variables of Internet use, mental health, social support, and family resilience were all regarded as continuous variables, so we first adopted the OLS model for analysis and included all control variables in the model. Secondly, to further analyze the robustness of the total effect, we also used quantile regression for comparison. Finally, we adopt the PROCESS model with 5,000 bias-corrected bootstrap samples and 95% confidence intervals to validate the chain mediation model and hypotheses. It is considered statistically significant when the effect does not include 0 in the 95% confidence interval (that is, the upper interval and the lower interval have the same sign). All data analyzes in our study were conducted using SPSS 26.0 and Mplus 8.0.

## Results

### Common method bias, validity and reliability

Before conducting regression analysis to test the research hypotheses, we first examined this study’s common method bias, validity, and reliability. To mitigate the influence of common method bias on the research findings, we employed two approaches: procedural control and statistical control. For procedural control, the research team followed strict procedures in data collecting, including anonymizing the questionnaires, keeping personal information confidential, and explaining the purpose of the survey to the respondents. In addition, different coding directions were designed in the questionnaire. In terms of statistical control, we used the Harman single-factor test to test common method bias. The findings revealed that the initial common factor accounted for 39.08% of the variance, falling below the critical threshold of 50% (Hair et al., 2014). This suggests that our study did not suffer from significant common method bias.

To assess validity, we conducted a confirmatory factor analysis to calculate the standardized factor loadings for all items in the three scales, and the results are shown in Table 1. Firstly, the Bartlett sphericity test statistics for the three scales were significant at the 0.1% level, and the KMO values exceeded 0.7, meeting the requirements for factor analysis. Secondly, the standardized factor loads for all items in the three scales ranged from 0.727 to 0.960, all surpassing the threshold value of 0.5 (Hair et al., 2014). Lastly, the average variance extraction value (AVE) for the three scales exceeded 0.5, indicating strong convergence validity. Additional calculations revealed that the square roots of the AVE values were all above 0.7, surpassing the correlation coefficient (refer to Table 2), which suggests strong discriminant validity.

**Table 2:** Results of the correlation analysis.

	Mental health	Internet use	Social support	Family resilience
Mental health	1.000			
Internet use	0.070^*^	1.000		
Social support	0.233^***^	0.151^***^	1.000	
Family resilience	0.233^***^	0.191^***^	0.576^***^	1.000
Mean	61.130	1.295	63.160	66.245
SD	17.515	1.429	20.590	21.202

*p < 0.1; **p < 0.05; ***p < 0.01.

Regarding reliability, we utilized composite reliability (CR) to assess the reliability of the three scales in our study. As indicated in Table 1, the composite reliability scores for all three scales surpass the recommended value of 0.7. Thus, the scales employed in our study demonstrate high reliability.

### Descriptive statistics

The results in Table 2 display the descriptive statistics for the core variables in our study, including means, standard deviations, and correlation coefficients. The findings indicate significant correlations among the core variables. Specifically, mental health is positively correlated with Internet use (*r* = 0.070, *p* < 0.1), social support (*r* = 0.233, *p* < 0.01), and family resilience (*r* = 0.233, *p* < 0.01). Internet use is also positively correlated with social support (*r* = 0.151, *p* < 0.01) and family resilience (*r* = 0.191, *p* < 0.01). Furthermore, social support shows a positive correlation with family resilience (*r* = 0.576, *p* < 0.01).

### Hypothesis testing

We first examined the total effect between Internet use and mental health. The regression results in Table 3 show that when all control variables are included, there is a significant positive relationship between Internet use and mental health (ß = 1.524, *p* < 0.05; Model 1). Additionally, we conducted quantile regression to evaluate the relationship between Internet use and different levels of mental health. The findings reveal that Internet use has a significant positive relationship with mental health at the 0.25, 0.5, and 0.9 percentiles (ß = 1.370, *p* < 0.05, Model 1.1; ß = 1.915, *p* < 0.01, Model 1.2; ß = 1.829, *p* < 0.05, Model 1.3), further verifying the robustness of the total effect.

**Table 3:** Total effect model regression results.

	Model 1	Model 1.1	Model 1.2	Model 1.3
	MH	MH (0.25)	MH (0.5)	MH (0.9)
**Predictor**				
Internet use	1.524^**^	1.370^**^	1.915^***^	1.829^**^
	(0.521)	(0.648)	(0.707)	(0.785)
**Control variables**				
Gender	6.451^***^	6.783^***^	8.883^***^	7.546^***^
	(1.334)	(1.661)	(1.812)	(2.010)
Age	0.265^***^	0.276^***^	0.287^***^	0.246^***^
	(0.062)	(0.077)	(0.084)	(0.093)
Education level	0.539	0.374	0.892	0.445
	(0.541)	(0.674)	(0.735)	(0.815)
Physical health status	3.144^***^	3.214^***^	3.454^***^	2.284^**^
	(0.640)	(0.796)	(0.868)	(0.963)
**Marriage status (single)**				
Married	−4.107^**^	−3.956^*^	−4.458^*^	−0.356
	(1.929)	(2.402)	(2.620)	(2.906)
Divorced	−1.379	−1.222	−2.536	−1.511
	(2.484)	(3.092)	(3.372)	(3.741)
Widowed	−3.997	−5.863^*^	−5.143	2.945
	(2.504)	(3.116)	(3.399)	(3.771)
Politics status	−5.767^*^	−3.749	−7.750^*^	−8.917^**^
	(2.936)	(3.655)	(3.987)	(4.423)
Average family income	3.230^***^	2.131^*^	3.031^**^	2.608^*^
	(0.944)	(1.175)	(1.282)	(1.422)
Social contact	2.021^***^	0.500	1.055	−3.474^*^
	(0.698)	(1.714)	(1.869)	(2.074)
Current residence	−0.449	1.638^*^	2.835^***^	1.954^*^
	(1.377)	(0.869)	(0.948)	(1.051)
Constant	−3.968	−4.191	−10.225	25.368^*^
	(10.134)	(12.614)	(13.759)	(15.265)
*N*	765	765	765	765
*R*^2^/Pseudo *R*^2^	0.358	0.067	0.085	0.076

Standard errors in parentheses; ^*^p < 0.1; ^**^p < 0.05; ^***^p < 0.01; MH, Mental health.

Hypothesis 1 assumes that there is a positive correlation between Internet use and the mental health of social assistance recipients. The regression results in Table 4 show that the coefficient of Internet use is positive and statistically significant (ß = 1.049, *p* < 0.05; Model 4), supporting hypothesis 1.

**Table 4:** Regression results of chain mediation model.

	Model 2	Model 3	Model 4
	SS	FR	MH
**Predictor**			
Internet use	1.796^***^	1.078^**^	1.049^**^
	(0.614)	(0.527)	(0.509)
**Mediator**			
Social support		0.523^***^	0.095^***^
		(0.031)	(0.035)
Family resilience			0.151^***^
			(0.035)
**Control variables**			
Gender	0.418	−2.042	6.686^***^
	(1.572)	(1.343)	(1.294)
Age	0.005	−0.004	0.265^***^
	(0.073)	(0.062)	(0.060)
Education level	0.269	0.971^*^	0.346
	(0.638)	(0.545)	(0.525)
Physical health status	1.003	−0.037	2.975^***^
	(0.754)	(0.644)	(0.620)
**Marriage status (single)**			
Married	3.148	9.683^***^	−6.112^***^
	(2.273)	(1.944)	(1.901)
Divorced	0.747	2.475	−1.881
	(2.926)	(2.500)	(2.407)
Widowed	2.729	3.415	−4.986^**^
	(2.950)	(2.521)	(2.429)
Politics status	7.085^**^	0.969	−7.146^**^
	(3.460)	(2.963)	(2.851)
Average family income	3.154^***^	1.616^*^	2.438^***^
	(1.112)	(0.955)	(0.921)
Social contact	6.262^***^	2.722^***^	0.521
	(0.822)	(0.729)	(0.708)
Current residence	0.145	−0.433	−0.409
	(1.622)	(1.386)	(1.333)
Constant	2.998	0.208	−4.521
	(11.939)	(10.198)	(9.812)
*N*	765	765	765
*R* ^2^	0.345	0.631	0.430

Standard errors in parentheses; ^*^p < 0.1; ^**^p < 0.05; ^***^p < 0 0.01; SS, Social support; FR, Family resilience; MH, Mental health.

Our second hypothesis proposed that social support acts as a mediator between Internet use and mental health. In Model 2, we included Internet use and control variables, and the regression results showed that Internet use was positively correlated with social support (ß = 1.796, *p* < 0.01; Model 2). Model 4 further incorporated Internet use, social support, family resilience, and all control variables. The results show that both Internet use (ß = 1.049, *p* < 0.05; Model 4) and social support (ß = 0.095, *p* < 0.01; Model 4) have a significant positive relationship with mental health. This suggests that social support acts as a mediating variable, supporting Hypothesis 2.

Hypothesis 3 speculates that family resilience is a mediating variable between Internet use and mental health. Our analysis in Model 3 included Internet use, social support, and control variables, revealing that there is a significant positive relationship between Internet use and family resilience (ß = 1.078, *p* < 0.05; Model 3). Furthermore, in Model 4, both Internet use and family resilience are significantly and positively related to mental health, confirming the mediating role of family resilience. In conclusion, hypothesis 3 is verified.

For hypothesis 4, we used the PROCESS model for verification. Specifically, we conducted 5,000 Bootstrap samplings to further examine the total, direct, and indirect effects between Internet use and mental health. If the 95% confidence interval does not contain 0, the effect is considered statistically significant. Results from Table 5 confirmed that Internet use is significantly positively correlated with mental health, which again validates hypothesis 1. In addition, the total indirect effect coefficient is 0.475 (Boot SE = 0.163, 95% CI = [0.198, 0.839]), and the 95% confidence interval does not include 0, indicating that the total indirect effect is significant. Among them, the path coefficient using social support as the mediating variable is 0.171 (Boot SE = 0.100, 95% CI = [0.017, 0.398]), and the path coefficient using family resilience as the mediator is 0.162 (Boot SE = 0.102, 95% CI = [0.001, 0.396]). The 95% confidence intervals of the two coefficients do not include 0, indicating that they are statistically significant. Hypothesis 2 and hypothesis 3 are verified again. Finally, the path coefficient using social support and family resilience as chain mediating variables is 0.141 (Boot SE = 0.064), and the 95% confidence interval is [0.038, 0.285], indicating that the chain mediation is established, supporting hypothesis 4.

**Table 5:** Non-standardized chain mediation analysis results.

Paths	Effect	Boot SE	LLCI	ULCI
**Total effect**				
IV → DV	1.524	0.521	0.501	2.547
**Direct effect**				
IV → DV	1.049	0.509	0.050	2.048
**Indirect effect**				
Total	0.475	0.163	0.198	0.839
IV → Social support→DV	0.171	0.100	0.017	0.398
IV → Family resilience→DV	0.162	0.102	0.001	0.396
IV → Social support→Family resilience→DV	0.141	0.064	0.038	0.285

IV, Internet use; DV, mental health; Bootstrap resample = 5,000; Control variables are included in the analysis.

## Discussion

### Summary of findings

This study mainly explores the relationship between Internet use and mental health and its specific mechanism. Data analysis of 765 Chinese social assistance recipients shows that there is a significant positive relationship between Internet use and mental health, and that perceived social support and family resilience play a mediating role in the relationship between Internet use and mental health, respectively. Moreover, social support and family resilience can act as chain mediating variables in this process. Specifically, Internet use can enhance social support, which in turn improves family resilience, ultimately leading to an improvement in the mental health of social assistance recipients. Our study also has several theoretical and practical implications, which will be discussed in the next section.

### Theoretical implications

Internet use is becoming more and more common in our daily life, study, and work. Scholars have conducted a lot of research on the relationship between Internet use and mental health. However, few studies have discussed the relationship between Internet use and mental health among social assistance recipients. In addition, there is little discussion on the possible chain mediation mechanism between Internet use and mental health through social support and family resilience. To fill these research gaps, we take the impact of Internet use on the mental health of social assistance recipients as an important research issue and use the survey data of Chinese social assistance recipients for empirical analysis. This study has the following theoretical implications.

Firstly, this study explores the relationship between Internet use and the mental health of social assistance recipients. Although a large number of studies have confirmed the positive relationship between Internet use and mental health, some studies have drawn different conclusions based on different study groups (Ma and Sheng, 2023; Du et al., 2024). To examine whether Internet use is positively related to the mental health of social assistance recipients, social support, family resilience, and control variables were included in the model. The results show that there is a significant positive correlation between Internet use and mental health, which is consistent with some research findings (Han and Zhao, 2021; Chen et al., 2023). Therefore, this study confirmed the positive relationship between Internet use and the mental health of social assistance recipients.

Secondly, based on Mindsponge Theory and COR Theory, this study revealed the chain mediation mechanism between Internet use and the mental health of social assistance recipients. First, when facing difficulties, social assistance recipients can improve their information accessibility through Internet use to obtain more information related to social support, thereby internalizing this information through the benefit–cost evaluation process to update their core values and improve their perceived social support level. At the same time, Internet use can also help recipients replenish various resources to alleviate the resource loss caused by family stress events, thereby improving their social support level. Second, the increase in perceived social support can enhance their confidence in development and enrich their available resources, thereby enhancing their ability to solve difficulties and improving their family resilience. Finally, family resilience, as an important trait for coping with stressful situations, helps to alleviate the stress response of social assistance recipients caused by the loss of resources and ultimately helps to improve their mental health. Therefore, we have expanded the research on Internet use and mental health and confirmed the possible mediation mechanism.

Last but not least, this study uses Chinese social assistance recipients as the research group, and the conclusions drawn may differ from those in Western contexts due to China’s specific cultural background. The cultural values centered on family ties may make Chinese social assistance recipients first seek help from their relatives, neighbors, and other groups when they face difficulties, and the rise of various social media and Internet assistance platforms (such as online crowdfunding platforms) provides an opportunity for these groups in distress to seek help and obtain resources from a wider social group. The cultural concept of “when disaster struck, help came from all sides “rooted in the collectivist values of Chinese society enables Chinese social assistance recipients to better internalize this information based on their core values when using the Internet to obtain social support information and resources. This will help them to improve their perceived level of social support to a greater extent, enhance family resilience, and thus better solve difficulties and maintain a high level of mental health.

### Practical implications

Considering the positive relationship between Internet use and the mental health of social assistance recipients and the chain mediation mechanism of social support and family resilience, this study has some practical implications.

To begin with, relevant departments should continue to guide social assistance recipients to use the Internet reasonably and give full play to the positive role of Internet use. Our research found that although Internet use is positively related to mental health, the Internet use frequency (mean value is 1.295) and education level (mean value is 2.252) of social assistance recipients are generally low, which is not conducive to maximizing the positive effects of Internet use. Therefore, the government and public welfare organizations should increase digital skills training for social assistance recipients and moderately improve their frequency of Internet use. At the same time, they should help them strengthen their connections with the outside world through the Internet and quickly obtain the information and resources they need to cope with difficulties. In addition, relevant companies should develop more easy-to-use and practical apps to help more social assistance recipients benefit from Internet use.

Subsequently, social support and family resilience are crucial to the mental health of social assistance recipients. Relevant departments need to prioritize the mental well-being of social assistance recipients. In addition to providing material support to these families, efforts should be made to increase their social support by strengthening connections with relatives, friends, colleagues, community leaders, and other members of their social network. Social workers and other professionals should also conduct social casework, social group work, and social community work to enhance the resilience of these families in need.

### Limitations and future directions

First of all, the sample of this study comes from social assistance recipients in China. Due to differences in culture and the level of internet development, their understanding of internet use and mental health may differ from social assistance recipients in other countries. In future studies, more relevant data from other countries are needed for comparison.

Secondly, we did not differentiate social assistance recipients by age group. Is there a positive relationship between Internet use and the mental health of social assistance recipients of different age groups? Is there a difference in the strength of this correlation? Are the mechanisms the same? These issues need to be further explored in future studies.

Thirdly, in the Mindsponge Theory, cultural values (i.e., information) and trust have important influences on people’s information processes. Among them, trust plays a key role in determining the absorption and exclusion of information. Specifically, trust is a mechanism for people to evaluate the benefits and costs of current information based on existing core values, which can help people make quick judgments about information. Therefore, the higher the degree of trust in information, the higher the degree of acceptance of information by people’s core values (Nguyen et al., 2022), which in turn affects people’s psychological and behavioral processes. However, due to the limited accessibility of research data, our research failed to fully examine the influence of different types of information, trust, and other factors in shaping the core values of social assistance recipients and the relationship between Internet use, social support, family resilience, and mental health. In future research, it is necessary to include factors such as the level of social trust of social assistance recipients and the type of information into the analysis to examine the possible influence of trust level and type of information received on the relationship between Internet use and mental health.

Lastly, our study used the average frequency of several Internet activities as independent variables to examine the relationship between Internet use and the mental health of social assistance recipients. However, there are differences in different types of Internet use, which may have different relationships with the mental health of social assistance recipients. In future studies, it is necessary to further explore the differences in the relationship between different types of Internet use and the mental health of social assistance recipients.

## Conclusion

This study examined the relationship between Internet use, social support, family resilience, and mental health among Chinese social assistance recipients. Data analysis results confirm that Internet use was positively correlated with the mental health of social assistance recipients, and that social support and family resilience played mediating roles between Internet use and mental health, respectively. In addition, social support and family resilience can act as chain mediators between Internet use and mental health. Eventually, our research findings can help relevant departments recognize the positive relationship between Internet use, social support, and family resilience and the mental health of social assistance recipients, and enhance them through relevant measures to improve the mental health of social assistance recipients.

## Data Availability

The original contributions presented in the study are included in the article/supplementary material, further inquiries can be directed to the corresponding author.
